# Eosinophil/Monocyte Ratio Combined With Serum Thyroid Hormone for Distinguishing Graves' Disease and Subacute Thyroiditis

**DOI:** 10.3389/fendo.2020.00264

**Published:** 2020-05-08

**Authors:** Yongbin Hu, Diyi Zhou, Jiawei Chen, Pengfei Shan

**Affiliations:** ^1^Department of Endocrinology, Hangzhou Red Cross Hospital, Hangzhou, China; ^2^Department of Endocrinology, The Second Affiliated Hospital of Zhejiang University School of Medicine, Hangzhou, China

**Keywords:** Graves' disease, subacute thyroiditis, triiodothyronine, thyroxine, eosinophils

## Abstract

**Background:** Thyrotoxicosis is commonly classified into several entities according to different etiologies. Identifying the causes of thyroid dysfunction is critical for the subsequent selection of treatment. The free triiodothyronine to free thyroxine ratio (fT_3_/fT_4_) is widely used but is still a controversial diagnostic measurement.

**Methods:** A total of 290 patients including 141 healthy control subjects, 86 patients with untreated Graves' disease (GD,) and 63 patients with subacute thyroiditis (SAT) were enrolled in the study. The main aim was to evaluate the diagnostic value of different indexes from serum testing including fT_3_, fT_4_, eosinophils (Eo) and monocytes (Mo). The diagnostic performance of multiple indexes was evaluated separately using receiver operating characteristic curve analysis.

**Results:** Sensitivities and specificities of fT_4_/fT_3_, Mo/Eo ratios and Mo/Eo ratio + fT_4_/fT_3_ for diagnosing GD were 80.23 and 88.89, 82.56 and 60.32, and 74.4 and 87.3 with cut-off values of ≤ 2.841, ≤ 8.813 and >0.644, respectively. An equation of combined indicators including Mo, Eo, fT_3_, and fT_4_ data was developed to calculate a probability value and among all indexes studied the indicator combination formula gave the best diagnostic value, reaching sensitivity and specificity of 89.53 and 90.48%, respectively, with an optimum cut-off value at 0.561 for GD diagnosis.

**Conclusion:** Compared to regular indexes (fT_4/_fT_3_ and Mo/Eo), a newly developed indicator combination formula provided a higher prediction probability and may serve as a simple, cost-effective tool for differentiating GD from SAT patients, especially in undeveloped regions of China.

## Introduction

Thyrotoxicosis is an excess of thyroid hormones in the blood caused by a common thyroid dysfunction. Thyroiditis disorders can be classified in several ways, based on different etiologies, different pathologies or according to different clinical presentations (1). Graves' disease (GD) and subacute thyroiditis (SAT) are two of the most common etiologies of thyrotoxicosis, along with others including toxic adenoma and multinodular goiter (2). SAT is an inflammatory disorder of the thyroid. Graves' disease is an autoimmune thyroid disease in which thyroid hormones are overproduced and secreted in excessive amounts (3), and is attributable to immunoglobulins that activate the thyroid-stimulating hormone (TSH) receptor of follicular cells (4). GD is usually diagnosed on the basis of clinical findings and laboratory tests showing high values of the free triiodothyronine (fT_3_) to free thyroxine (fT_4_) ratio (fT_3_/fT_4_), low levels of TSH, and/or high radioactive iodine uptake (RAIU) as well as TSH-receptor stimulating antibodies. In Graves' disease fT_3_ and fT_4_ serum levels are usually higher compared with subacute thyroiditis and patients with controlled GD are those with normal or mildly elevated thyroid hormone levels (fT_4_ <30 pmol/L). Indeed, while urgent thyroidectomy can be performed with a rapid control of thyrotoxicosis, severe thyrotoxicosis can be treated pharmacologically (5, 6). Diagnosis of SAT in the thyrotoxic phase is made on the basis of the clinical features of neck pain and swelling, tenderness and fever, and laboratory findings of increased C-reactive protein (CRP), fT_4_ and fT_3_, and/or low RAIU.

Hyperthyroidism patients with GD must be treated with antithyroid drugs, radioisotope therapy or surgery (4), which is totally different from patients with SAT. The purpose of treatment for SAT is to reduce pain and inflammation and to treat any hyperthyroidism. Anti-inflammatory drugs such as aspirin, ibuprofen or corticosteroids are used to control pain in mild cases of SAT (7).

Differentiation of destruction-induced thyrotoxicosis (painless or subacute thyroiditis) from GD is important for the selection of the correct therapy. GD but not SAT can be triggered by stressful events (8, 9) and albeit hypoechogenicity can be present in both, even though in GD the thyroid is diffusely hypoechoic, hypervascularization is generally not present in patients with SAT (10, 11). It is, however, often difficult to make this distinction without measurement of RAIU. In order to determine efficiently the cause of thyroid dysfunction, several studies have evaluated various indexes for differentiating GD from SAT. The serum thyrotropin (more frequently referred to as thyroid-stimulating hormone, TSH) level is the most sensitive and specific tool used for the diagnosis of primary thyroid dysfunction (2). A third-generation assay for circulating antibodies to the thyrotropin receptor (TRAb) has been developed, offering a sensitivity of 95 and 100% specificity, but mild GD patients might be TRAb negative (12). Serum fT_3_ levels in GD patients were significantly higher than in patients with painless thyroiditis and SAT (13). The free fT_3_/fT_4_ of patients with painless thyroiditis overlapped with those of patients with GD. However, for differentiating between these two disorders this ratio could only be applied when the fT_4_ value was high (14). Another study demonstrated that a fT_3_/fT_4_ ratio of > 4.4 (10^−2^ pg/ng) may help in differentiating the cause of thyrotoxicosis (2). In addition, an eosinophil/monocyte (Eo/Mo) ratio <0.2 and/or Eo/Mo ratio multiplied by fT_3_ <4.5 in untreated thyrotoxic patients have been proposed as laboratory signals of destruction-induced thyrotoxicosis, which can help to determine these two forms of thyrotoxicosis without needing the RAIU test (15). Other than individual and ratio indexes, the combination of multiple indexes may improve the sensitivity and specificity of diagnosis in the clinic.

In a large number of basic hospitals and physical examination sites, only thyroid functions such as fT_3_, fT_4_, and TSH levels are analyzed. If hyperthyroidism is confirmed, more diagnostic criteria (TRAb, erythrocyte sedimentation rate (ESR), thyroid ultrasound and RAIU) must be examined in order to identify the cause of hyperthyroidism, which costs more money and time for patients. In this retrospective study, we aimed to find a simple, highly sensitive and specific tool that could differentiate GD from SAT patients in China.

## Materials and Methods

### Patients

This was a retrospective study. All the subjects were from the physical examination center or outpatient clinic of Hangzhou Red Cross Hospital from 2016 to 2018. A total of 290 patients were enrolled and divided into 3 groups namely controls (141 subjects), GD (86 patients) and SAT (63 patients) groups. Subjects in the control group had normal thyroid function. Patients with GD were diagnosed as hyperthyroidism, positive TRAb response, ESR <30 mm/h and/or high RAIU. No anti-hyperthyroidism drugs were taken within 6 months after the initial diagnosis and the elevated fT_3_ and fT_4_ returned to normal or decreased for SAT patients. In addition, ESR > 30 mm/h and thyroid tenderness or ultrasound examinations suggesting hypoechoic masses, and/or a low RAIU were also used to characterize SAT patients in our study. All blood samples were collected before treatment. Patients who were pregnant or had severe cardiopulmonary and renal diseases were excluded. The hospital's scientific ethics committee approved the study. Written informed consent for participation was not required for this study in accordance with the national legislation and the institutional requirements.

### Measurements

Serum levels of fT_4_ and fT_3_ were measured by radioimmunoassay and anti-TSH receptor antibodies (TBII) were detected by radioreceptor assay (Bayer Cooperation Automated Chemiluminescence System, US). Peripheral leukocyte counts and the percentages of eosinophils and monocytes were measured using an automated leukocyte differential system (Total Hematology Management System NE-7000; Sysmex Co., Kobe, Japan).

### Statistically Analysis

All statistical analyses were performed using SPSS (SPSS Statistics for Windows, ver. 18.0, SPSS Inc., US). Descriptive data are shown as means ± SD for normally distributed parameters. *P* < 0.05 was considered to be a statistically significant difference. A chi-squared test was used for analysis of categorical variable (gender). One-way ANOVA was used to look for significant differences among the three groups for continuous parameters. Comparison between two groups with significant difference was tested using the SNK method. Comparison between two groups of continuous parameters that were normally distributed was conducted using a *t*-test. A receiver operating characteristic (ROC) curve analysis was performed to obtain the optimal cut-off values for all tested indexes used for the diagnosis of GD.

## Results

### Clinical Characteristics of Individuals

The clinical data of the study population are shown in Table 1. In the total of 86 GD patients (38.1 ± 16.1 years), 78% (67/86) were female with significantly higher fT_3_ and fT_4_ levels than the healthy controls (41.9 ± 9.6 years) and SAT groups (43.2 ± 9.4 years). Monocyte counts were also remarkably different among the three groups, being highest in the untreated GD group (10.0 ± 3.0), followed by SAT (7.6 ± 2.3) and the healthy control group (6.6 ± 2.1). Additionally, the fT_4/_fT_3_ and Mo/Eo ratios were also significantly different among all three groups, indicating that these indexes might serve to differentiate GD patients from SAT patients and healthy controls. In addition, there was no significant differences between males and females regarding Mo (%), Eo (%), fT_3_ (pmol/L), fT_4_ (pmol/L), fT_4_/fT_3_ and Mo/Eo in the 3 groups and the differences between GD and SAT patients did not show gender specifities (Supplementary Table 1).

**Table 1:** Clinical characteristics of the study population.

	**Healthy control**	**Untreated Graves' disease**	**Subacute thyroiditis**	***P*-value**
Gender (M/F)				
Male	49 (34.7%) ^a^	19 (22.1%) ^b^	9 (14.3%)^b^	0.005
Female	92 (65.3%)	67 (77.9%)	54 (85.7%)	
Age (years)	41.9 ± 9.6^a^	38.1 ± 16.1^b^	43.2 ± 9.4^a^	0.020
Mo (%)	6.6 ± 2.1^a^	10.0 ± 3.0^b^	7.6 ± 2.3^c^	<0.001
Eo (%)	2.2 ± 1.3^a^	2.0 ± 1.2^a^	0.9 ± 0.7^b^	<0.001
fT_3_ (pmol/L)	5.4 ± 0.7^a^	18.2 ± 7.9^b^	10.0 ± 5.5^c^	<0.001
fT_4_ (pmol/L)	17.3 ± 2.2^a^	44.5 ± 20.4^b^	32.9 ± 14.1^c^	<0.001
fT_4/_fT_3_	3.3 ± 0.4^a^	2.5 ± 0.6^b^	3.5 ± 0.8^c^	<0.001
Mo/Eo	4.3 ± 3.2^a^	7.0 ± 5.3^b^	15.9 ± 15.4^c^	<0.001

*The same letters indicate no statistically significant difference between the two groups, different letters indicate statistically significant differences between the two groups (a vs. a means P > 0.05, a vs. b means P < 0.05, a vs. c means P < 0.05, b vs. b means P > 0.05, b vs. c means P < 0.05). Eo, eosinophils; fT_3_, free triiodothyronine; fT_4_, free thyroxine; Mo, monocytes*.

### Analysis Based on Moderate and Severe Thyrotoxicosis Groups

Generally, fT_4_ is obviously elevated in thyrotoxicosis patients. Hence, GD and SAT patients in this study were classified into moderate and severe thyrotoxicosis entities according to the fT_4_ level (Table 2). All six indexes including Mo (%), Eo (%), fT_3_ (pmol/L), fT_4_ (pmol/L), fT_4_/fT_3_ ratio and Mo/Eo ratio were statistically analyzed for both GD and SAT patients. For moderate thyrotoxicosis patients (fT_4_ ≤ 30 pmol/L), five indexes, except fT_4_, showed high significance for distinguishing between these two etiologies. In severe thyrotoxicosis patients (fT_4_ > 30 pmol/L), all indexes showed significant differences between GD and SAT patients. Hence, our data implied that all four single indexes from regular lab tests and their ratios can be potentially employed as sensitive parameters for diagnosing the etiology of thyrotoxicosis, without any further time consuming and expensive testing. Therefore, we evaluated the diagnosis performance of these indexes and also different combination of indexes from serum testing in order to establish unequivocally the best indicator with the highest prediction probability for accurate diagnosis.

**Table 2:** Comparison of GD and SAT patients in moderate (fT_4_ ≤ 30 pmol/L) and severe (fT_4_ > 30 pmol/L) thyrotoxicosis entities.

	**Moderate** **fT**_****4****_ **≤** **30pmol/L**			**Severe** **fT**_****4****_ **>** **30 pmol/L**		
	**GD** ** (*n* = 26)**	**SAT** ** (*n* = 33)**	***P*-value**	**GD** **(*n* = 60)**	**SAT** **(*n* = 30)**	***P*-value**
fT_4_ (pmol/L)	25.2 ± 4.2	23.5 ± 4.8	0.156	52.8 ± 18.9	43.3 ± 13.7	0.008
Mo (%)	9.2 ± 3.0	7.7 ± 2.3	0.025	10.4 ± 2.9	7.6 ± 2.5	<0.001
Eo (%)	2.2 ± 1.6	0.9 ± 0.7	0.001	2.0 ± 1.1	0.8 ± 0.6	<0.001
fT_3_ (pmol/L)	10.3 ± 2.0	7.0 ± 1.7	<0.001	21.6 ± 7.1	13.3 ± 6.3	<0.001
fT_4/_fT_3_	2.5 ± 0.6	3.4 ± 0.6	<0.001	2.5 ± 0.6	3.5 ± 1.0	<0.001
Mo/Eo	5.9 ± 3.8	15.4 ± 12.2	<0.001	7.4 ± 5.8	16.4 ± 18.5	0.014

*Eo, eosinophils; fT_3_, free triiodothyronine; fT_4_, free thyroxine; GD, Graves' disease; Mo, monocytes; SAT, subacute thyroiditis*.

### Diagnostic Value of Individual and Combined Indexes in Patients With Untreated GD

**Figure 1:**
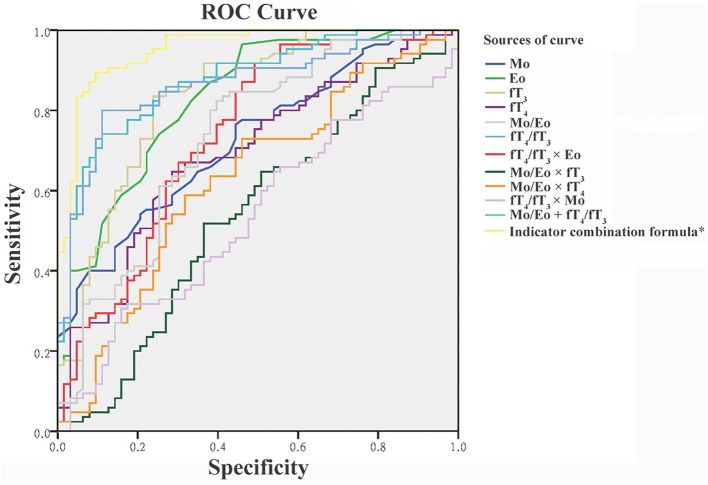
Receiver operating characteristic (ROC) curve for the discrimination of patients with Graves' disease from healthy controls and patients with subacute thyroiditis. *The detailed indicator combination formula is presented in the text. Eo, eosinophils; fT_3_, free triiodothyronine; fT_4_, free thyroxine; Mo, monocytes.

To obtain the optimal diagnostic cut-off value of all proposed indexes, ROC curve analysis of all untreated thyrotoxicosis patients and healthy control population were performed as shown in Figure 1. Among four individual indexes (Mo, Eo, fT_3_, and fT_4_), fT_3_ with an optimal cut-off at 10.770 showed the highest sensitivity and specificity of 82.56 and 76.19%, respectively. In terms of ratio indexes, the fT_4/_fT_3_ ratio was obviously better than the Mo/Eo ratio with 80.23% sensitivity and 88.89% specificity, which was also better than fT_3_. A combination indicator including Mo, Eo, fT_3_ and fT_4_ data calculated as indicator combination formula for GD diagnosis: exp (6.8009 + 0.5529 × Mo + 0.9628 × Eo - 0.3765 × fT_3_ + 0.1694 × fT_4_ - 4.4571 × fT_3_/fT_4_ - 0.0871 × Mo/Eo)/[1 + exp (6.8009 + 0.5529 × Mo + 0.9628 × Eo - 0.3765 × fT_3_ + 0.1694 × fT_4_ - 4.4571 × fT_3_/fT_4_ - 0.0871 × Mo/Eo)] with an optimum cut-off value at 0.561 led to the best diagnostic value, reaching an accuracy of 89.93% (Table 3).

**Table 3:** Diagnostic evaluation of cut-off levels of various indexes in untreated Graves' disease.

	**Cut-off**	**Sensitivity (%)**	**Specificity (%)**	**Accuracy (%)**
Mo	>9.500	53.49	79.37	64.43
Eo	>0.600	96.51	53.97	78.52
fT_3_	>10.770	82.56	76.19	79.87
fT_4_	>34.000	63.95	71.43	67.11
fT_4/_fT_3_	≤ 2.841	80.23	88.89	83.89
Mo/Eo	≤ 8.813	82.56	60.32	73.15
Mo/Eo × fT_3_	≤ 86.332	52.33	63.49	57.05
Mo/Eo × fT_4_	≤ 238.183	58.14	68.25	62.42
fT_4/_fT_3_ × Mo	≤ 17.540	30.23	84.13	53.02
fT_4/_fT_3_ × Eo	>2.076	91.86	49.21	73.83
Mo/Eo +fT_4_/fT_3_	>0.644	74.40	87.30	79.87
Indicator combination formula^*^	>0.561	89.53	90.48	89.93

* *The detailed indicator combination formula is presented in the text. Eo, eosinophils; fT_3_, free triiodothyronine; fT_4_, free thyroxine; Mo, monocytes*.

Other potential indexes including Mo/Eo × fT_3_, Mo/Eo × fT_4_, fT_4/_fT_3_ × Mo, fT_4/_fT_3_ × Eo and Mo/Eo ratio + fT_4_/fT_3_ were also analyzed (Table 3). But neither of them showed a competitive value compared to the fT_4/_fT_3_ ratio and a combination indicator. Using the optimal cut-off value based on ROC analysis, the positive predictive values (PPVs) and negative predictive values (NPVs) were also calculated, as shown in Table 4. The indicator combination formula exhibited the highest PPV of 92.77% compared to the fT_4_/fT_3_ ratio, Mo/Eo ratio, Mo/Eo × fT_3_, Mo/Eo × fT_4_, fT_4_/fT_3_× Mo, fT_4_/fT_3_ × Eo and Mo/Eo ratio + fT_4_/fT_3_. In addition, the positive likelihood ratio (PLR) of the fT_4/_fT_3_ ratio and the indicator combination formula were the highest, reaching 7.22 and 9.40, respectively.

**Table 4:** Positive value and likelihood ratio of cut-off levels of various indexes in untreated Graves' disease.

	**Cut-off value**	**PPV (%)**	**NPV (%)**	**PLR**	**NLR**
fT_4/_fT_3_	2.841	90.79	76.71	7.22	0.22
Mo/Eo	8.813	73.96	71.70	2.08	0.29
Mo/Eo × fT_3_	86.332	66.18	49.38	1.43	0.75
Mo/Eo × fT_4_	238.183	71.43	54.4	1.83	0.61
fT_4_/fT_3_ × Mo	17.540	72.22	46.90	1.90	0.83
fT_4/_fT_3_ × Eo	2.076	71.17	81.58	1.81	0.17
Mo/Eo + fT_4_/fT_3_	0.644	88.89	71.43	5.86	0.29
Indicator combination formula^*^	0.561	92.77	86.36	9.40	0.12

* *The detailed indicator combination formula is presented in the text. Eo, eosinophils; fT_3_, free triiodothyronine; fT_4_, free thyroxine; Mo, monocytes; NLR, negative likelihood ratio; NPV, negative predictive value; PLR, positive likelihood ratio; PPV, positive predictive value*.

## Discussion

Thyrotoxicosis occurs in ~2% of women and 0.2% of men, with thyrotoxicosis caused by GD commonly developing between the second and fourth decades of life (16). To treat thyrotoxicosis appropriately, determining the underlying cause of the disease is essential. Increased thyroid RAIU with diffused uptake on scanning and raised serum thyroid stimulating immunoglobulin may be present in GD patients. SAT is a destructive thyrotoxicosis that often occurs after a viral infection, in which thyroid follicles are destroyed and T_4_ is released into the blood, so the increase of T_4_/T_3_ levels are more obvious (17, 18). In order to establish a simple, cost-effective, and fast method for differentiating GD from SAT, we evaluated multiple indexes to differentiate between these two etiologies.

The fT_4/_fT_3_ ratio has been widely evaluated as the indicator for differentiating GD from thyrotoxicosis (2, 14, 15, 19). T_3_ is the active form of thyroid hormone and is deiodinated from T_4_ by thyroid type 1 or type 2 deiodinase (20), and only 20% circulating T_3_ is reportedly secreted by the thyroid in euthyroid individuals (21). However, thyroid hyperfunction leads to an increase of T_3_ levels other than increased T_4_ to T_3_ peripheral conversion, the majority converted from T_4_ deiodinated in the thyroid (22). The activity of type 1 and type 2 deiodinase is higher in the GD thyroid gland, leading to enhanced conversion from T_4_ to T_3_ (23). Hence T_3_ levels and the fT_4_/fT_3_ ratio in GD patients is obviously changed (24). However, the use of the fT_4/_fT_3_ ratio is still not a standard method in diagnosing the etiology of thyrotoxicosis due to controversial reports based on different patient populations. Our study has shown that an optimum cut-off value of the fT_4/_fT_3_ ratio at 2.841 provides a sensitivity of 80.23% and a specificity of 88.89%, which is not perfect but still highly acceptable.

Izumi et al. (1) proposed that GD might be related to a Th2-predominant condition which was underlined by frequent relapses of Graves' thyrotoxicosis after an allergic rhinitis attacks, which is a typical Th2 disease. Since patients suffering from Th2-dominant diseases often show increased levels of peripheral eosinophils, they focused particularly on peripheral eosinophils, which were increased in thyrotoxic patients with GD. In their study that included 111 untreated patients with thyrotoxicosis, the eosinophil percentage was significantly higher in GD (3.54 ± 4.18%) and lower in SAT (1.08 ± 1.03%) compared to healthy controls (2.26 ± 1.33%) (15). Also in the present study, the percentage of eosinophils was significantly higher in GD than in subacute thyroiditis cases (Table 1). But compared to healthy controls, Eo levels in subacute thyroiditis patients seemed to be reduced, without changes in the GD cases. In addition, since the Mo/Eo ratio was also determined by an increased Mo level in GD patients, the Mo/Eo ratio alone might not be a straightforward indicator for GD diagnosis. Although our findings suggest the fT_4/_fT_3_ ratio is superior to the Mo/Eo ratio for diagnosis, the diagnostic accuracy still did not meet our expectations. Hence, we introduced the combination indicator, which utilizes four parameters in one equation that can be easily obtained after a routine blood test. Interestingly, the specificity and sensitivity achieved from the combination indicator were fundamentally elevated to 89.53 and 90.48%, respectively (Table 3).

In economically underdeveloped regions of China, the economic efficiency ratio is often the primary focus of health inspectors. Although screening for ESR, TRAb, thyroid ultrasound and RAIU is valuable in identifying the causes of thyrotoxicosis, many patients who have elevated T_3_ and T_4_ are unwilling or do not have the time to undertake further testing. In the present study, we compared a multiple combination of four individual indexes and eventually found the best option for differentiating GD from SAT patients, with an approximately 90% accuracy. With our proposed prediction equation of the combination indicator, simply testing for fT_3_, fT_4_, Mo and Eo values can basically identify the etiology of thyrotoxicosis, which might be clinically relevant especially in basic-level hospitals or physical examination institutions. If combined with a simple inquiry [whether there is fever or neck pain (25, 26)] and palpation of the thyroid gland, the derived formula should serve to distinguish whether thyroid toxicosis is GD or SAT related.

A limitation of the study is that the included male population is too small for a gender-stratified analysis and the smoking status of the participants has not been evaluated.

## Conclusions

This study demonstrated that combining Mo, Eo, fT_3_, and fT_4_ values into one combination indicator equation was highly effective for differentiating GD from SAT. Our proposed logistic equation offers a simple, cost-effective tool for diagnosing the etiology of thyrotoxicosis patients, involving four parameters that can be easily obtained from a regular blood test, even in undeveloped regions of China.

## Data Availability Statement

The datasets generated and/or analyzed are available from the corresponding author on reasonable request.

## Ethics Statement

The studies involving human participants were reviewed and approved by Ethics committee of Hangzhou Red Cross Hospital. Written informed consent for participation was not required for this study in accordance with the national legislation and the institutional requirements.

## Author Contributions

YH was responsible for the conception and design of the study. YH, DZ, JC, and PS were responsible for acquisition and analysis of data, furthermore, DZ was in charge of statistical analysis. YH drafted the manuscript, YH and DZ revised and commented on the draft, and all authors read and approved the final version of the manuscript.

## Conflict of Interest

The authors declare that the research was conducted in the absence of any commercial or financial relationships that could be construed as a potential conflict of interest.

## Supplementary Material

Supplementary Table 1 Gender analyses of clinical characteristics of the study population.
