# Supplementary material for: Eosinophil/Monocyte Ratio Combined With Serum Thyroid Hormone for Distinguishing Graves' Disease and Subacute Thyroiditis
Source: Front Endocrinol (Lausanne). 2020 May 8;11:264. doi: 10.3389/fendo.2020.00264 (PMC7225255; doi:10.3389/fendo.2020.00264)
Supplement: Supplementary Table 1 — Gender analyses of clinical characteristics of the study population. [file Table_1.DOCX]

**Supplementary Table 1.** Gender analyses of clinical characteristics of the study population

|  | Healthy control | | | Untreated GD | | | | SAT | | | Male  GD *vs.* SAT | Female  GD *vs.* SAT |
| --- | --- | --- | --- | --- | --- | --- | --- | --- | --- | --- | --- | --- |
|  | Male  (N = 49) | Female  (N = 92) | *P*-value | Male  (N = 19) | Female  (N = 67) | *P*-value | Male  (N = 9) | | Female  (N = 54) | *P*-value | *P*-value | *P*-value |
| Age (years) | 38.73 ± 8.78 | 43.54 ± 9.59 | 0.003 | 37.21 ± 12.29 | 38.4 ± 17.14 | 0.778 | 38.78 ± 11.41 | | 43.94 ± 8.93 | 0.128 | 0.750 | 0.024 |
| Mo (%) | 6.88 ± 1.83 | 6.51 ± 2.16 | 0.308 | 10.05 ± 1.31 | 10.21 ± 3.19 | 0.837 | 8.43 ± 1.98 | | 7.48 ± 2.39 | 0.261 | 0.016 | < 0.0001 |
| Eo (%) | 2.35 ± 1.52 | 2.13 ± 1.21 | 0.367 | 2.73 ± 1.81 | 1.85 ± 0.95 | 0.055 | 0.51 ± 0.29 | | 0.89 ± 0.64 | 0.086 | < 0.0001 | < 0.0001 |
| fT_3_ (pmol/L) | 5.39 ± 0.61 | 5.38 ± 0.68 | 0.920 | 17.1 ± 9.16 | 18.5 ± 7.60 | 0.501 | 9.78 ± 5.25 | | 10.06 ± 5.56 | 0.887 | 0.035 | < 0.0001 |
| fT_4_ (pmol/L) | 17.4 ± 2.15 | 17.32 ± 2.20 | 0.852 | 51.99 ± 25.86 | 43.6 ± 18.71 | 0.119 | 33.03 ± 8.78 | | 32.51 ± 14.53 | 0.917 | 0.043 | 0.001 |
| fT_4/_fT_3_ | 3.25 ± 0.38 | 3.25 ± 0.46 | 0.934 | 2.72 ± 0.45 | 2.47 ± 0.54 | 0.073 | 4.03 ± 1.23 | | 3.40 ± 0.68 | 0.170 | 0.0003 | < 0.0001 |
| Mo/Eo | 4.31 ± 2.90 | 4.31 ± 3.37 | 0.990 | 5.33 ± 3.80 | 7.42 ± 5.56 | 0.065 | 21.23 ± 12.22 | | 14.99 ± 15.8 | 0.264 | 0.004 | 0.001 |

Note: Eo, eosinophils; fT_3_, free triiodothyronine; fT_4_, free thyroxine; GD, Graves’ disease; Mo, monocytes; SAT, subacute thyroiditis.
